# Perioperative ROTEM® evaluation in a patient affected by severe VII factor deficiency undergoing microvascular decompression craniotomy for hemifacial spasm

**DOI:** 10.1007/s10877-024-01183-w

**Published:** 2024-06-08

**Authors:** Michele Introna, Morgan Broggi, Paolo Ferroli, Donato Martino, Carmela Pinto, Monica Carpenedo, Marco Gemma

**Affiliations:** 1 Neurointensive Care Unit, Department of Neurosurgery, Fondazione IRCCS Istituto Neurologico Carlo Besta, Milano, Italy; 2 Department of Anesthesiology, University of Groningen, University Medical Center Groningen, Groningen, The Netherlands; 3 Department of Neurosurgery, Fondazione IRCCS Istituto Neurologico Carlo Besta, Milan, Italy; 4 Hematology Unit, ASST Fatebenefratelli-Sacco, Ospedale L. Sacco, Polo Didattico Università degli Studi di Milano, Milano, Italy

**Keywords:** Thromboelastography, Microvascular decompression, ROTEM, Factor VII deficiency, Rare bleeding disorders, Hemifacial spasm

## Abstract

The potential use of TEG/ROTEM® in evaluating the bleeding risk for rare coagulation disorders needs to be assessed, considering the common mismatch among laboratory tests and the clinical manifestations. As a result, there is currently no published data on the use of viscoelastic tests to assess coagulation in FVII deficient patients undergoing elective neurosurgery. We describe the case of a patient affected by severe FVII deficiency who underwent microvascular decompression (MVD) craniotomy for hemifacial spasm (HFS). The ROTEM® did not show a significant coagulopathy according to the normal ranges, before and after the preoperative administration of the recombinant activated FVII, but a substantial reduction in EXTEM and FIBTEM Clotting Times was noted. The values of coagulation in standard tests, on the contrary, were indicative of a coagulopathy, which was corrected by the administration of replacement therapy. Whether this difference between ROTEM® and standard tests is due to the inadequacy of thromboelastographic normal ranges in this setting, or to the absence of clinically significant coagulopathy, has yet to be clarified. Neurosurgery is a typical high bleeding risk surgery; additional data is required to clarify the potential role for thromboelastographic tests in the perioperative evaluation of the FVII deficient neurosurgical patients.

## Introduction

Factor VII deficiency is the most frequent among the rare congenital coagulation disorders, with an estimated prevalence of around 1:300 000 in European countries [1, 2]. The classification proposed by the International Society on Thrombosis and Hemostasis considers an inherited deficit as “severe” if FVII activity is below 10% [3, 4]. Nevertheless, up to one-third of the individuals affected by inherited FVII deficiency do not suffer from any risk of significant bleeding [5]. Given its rarity and the heterogeneity of the clinical presentations, there are very few resources for guiding clinicians who deal with FVII deficient surgical patients. According to the largest study on FVII deficiency, a significant predictor for using recombinant activated FVII (rFVIIa) replacement therapy (RT) in surgical patients is prior major bleeding [5]. However, it is still matter of debate whether RT for FVII deficient patients should be limited to already bleeding patients, and no clear guidelines exist to help clinicians in the risk-benefit assessment [6].

Thromboelastography (TEG) and ROtational ThromboElastoMetry(ROTEM®), are non-invasive, point-of-care tests that allow an assessment of the clot formation and dissolution [7]. They are assumed to be superior to screening coagulation tests, such as PT and APTT, at least in acute traumatic coagulopathy, as capable of defining the contribution of blood cells and providing information about the clot stability.

The potential role of TEG/ROTEM® in assessing bleeding risk for FVII deficient patients needs evaluation, as evidence in rare bleeding disorders undergoing invasive procedures is limited to a few case reports [8–13]. However, there is no data in the literature on the possibility that viscoelastic tests are superior to conventional laboratory tests in quantifying the risk of bleeding for neurosurgical patients.

## Case presentation

We describe the case of a 79-year-old male with a history of *miastenia gravis* previously treated with thymectomy and a recent femoral aneurysm repair, after which he received chronic antiplatelet therapy. The patient also has been suffering from left facial hemispasm (HFS), with a temporary and unsatisfactory response to conservative treatment with botulinum toxin. The brain magnetic resonance (MR) documented a probable neurovascular conflict at the Root Entry Zone (REZ) of the VII-VIII cranial nerve complex, in the left cerebellopontine angle.

After multidisciplinary team assessment, a microvascular decompression (MVD) craniotomy was recommended, with the indication to stop acetylsalicylic acid seven days before the operation. MVD for HFS is generally considered a low-risk procedure, with 0.3% mortality according to the National Surgical Quality Improvement Program Analysis [14]. Nevertheless a 11% incidence of intraoperative bleeding and a 4% incidence of postoperative bleeding were reported [15]. The potential risk for catastrophic consequences in case of intracranial bleeding in the posterior fossa, although rare, prompt clinicians to carefully assess patients undergoing MVD operations.

During the routine preoperative screening the patient had an INR of 2.56 (normal 0.8–1.25), with normal activated partial thromboplastin ratio (aPTT ratio) of 1.0 (normal 0.79–1.27), normal platelets count (144 × 10^3/microliter), normal fibrinogen levels (332 mg/dl). All the other values were within the physiologic range, including renal and liver function screening tests.

The case was referred to the consultant hematologist, who diagnosed a FVII deficiency, with a measured FVII: C plasma level of 4% (normal 50–150%). The patient also referred a negative history for complications after the previous surgeries and no significant bleeding episodes throughout his life, with an ISTH-SSC Bleeding Assessment Tool of 0 [16]. The patient had no recollection, and no documents were in his possession, of the administration of RT for previous surgeries.

The strategy agreed between the anesthesiologist, the neurosurgeon, and the consultant hematologist, was to correct the FVII deficiency preoperatively with rFVIIa (NovoSeven®, NovoNordisk, Denmark), as authorized by the European Medicine Agency [17] because in our Institution the non-activate, plasma-derived FVII is not available. To limit the known potential thrombotic risk of the rFVII, we established to give just the prophylactic dose before surgery and to repeat the administration after the procedure just in case of bleeding.

**Fig. 1:**
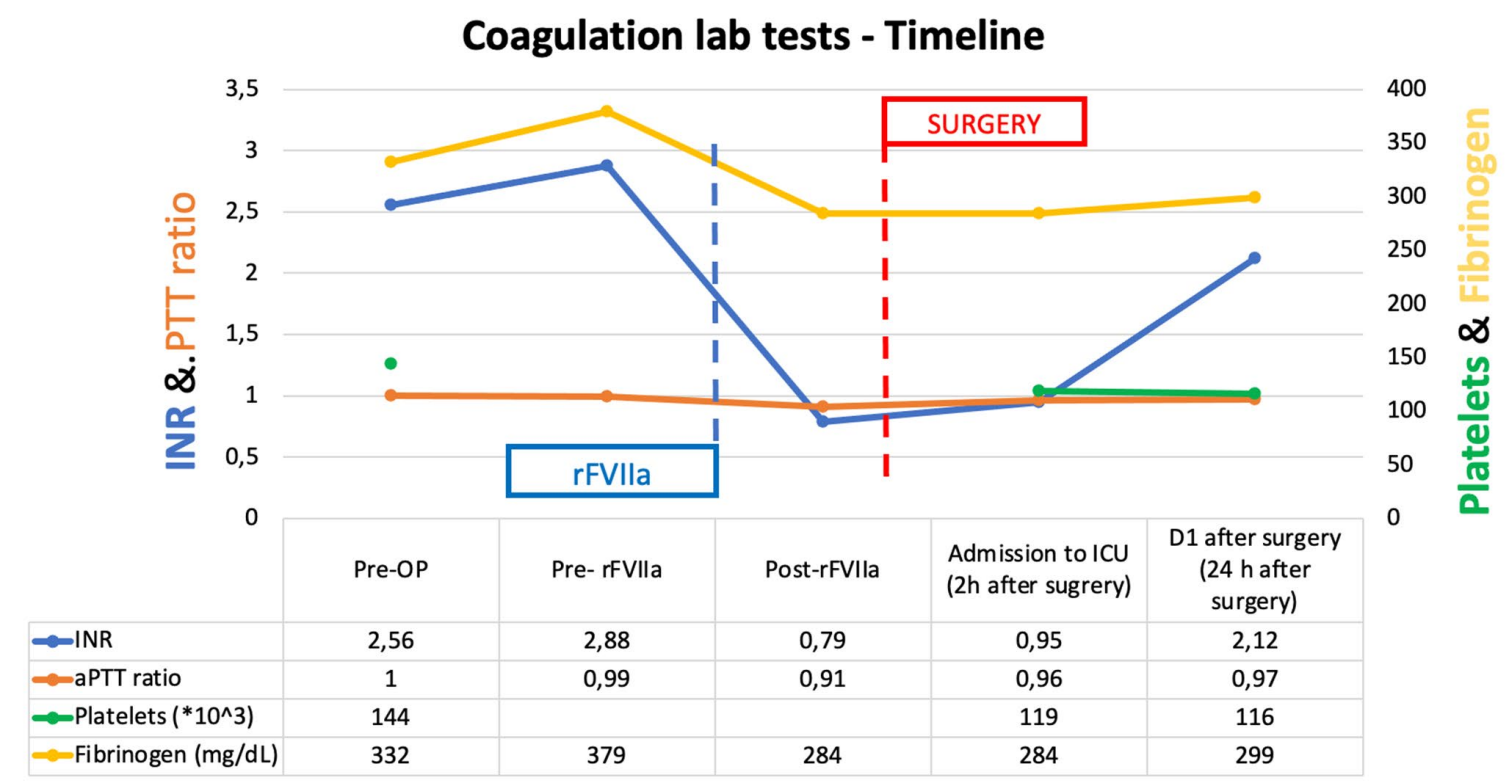
Timeline of perioperative conventional coagulation lab tests. The surgery procedure was performed after the infusion of rFVIIa. rVIIa = (recombinant activated VII factor, INR = international normalized ratio, aPTT = activated partial thromboplastin time

The trend of conventional coagulation lab tests before and after surgery is presented in Fig. 1.

**Fig. 2:**
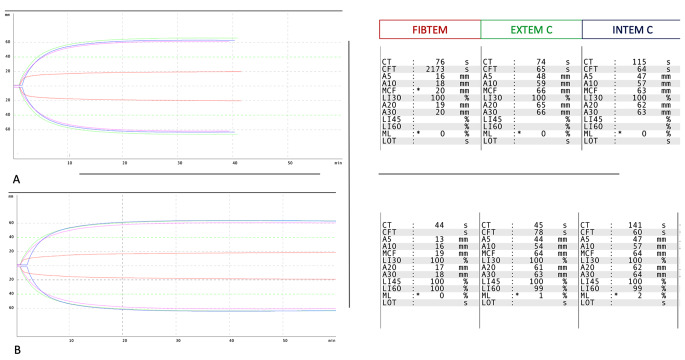
ROTEM® overlapping traces and values, repeated before (**A**) and after (**B**) rFVIIa infusion. CT—clotting time (s), CFT—clot formation time (s), A5—clot strength value in time of 5 min from CT (mm), A10—clot strength value in time of 10 min from CT (mm), A20—clot strength value in time of 20 min from CT (mm), A30—clot strength value in time of 30 min from CT (mm), MCF—maximum clot firmness (mm), LI30—lysis index 30 min after clotting time (%), (%), LI45—lysis index 45 min after clotting time (%), LI60—lysis index 60 min after clotting time (%), ML—maximum clot lysis (%)

The INR corrected after 15 min from the infusion of a bolus of 30 mcg/kg of rFVIIa, administered 30 min before surgery, according to the indications of the summary of product characteristics. All the other values did not change perioperatively. The MVD procedure was uneventful, and no signs of abnormal bleeding were reported by the surgeon; the patient experienced immediate relief of the HFS, without any complication or new neurological deficit. A CT scan was performed 1 and 24 h after surgery, showing no signs of bleeding. As expected, the INR raised to abnormal values already in 24 h, but - in the absence of any evidence of bleeding - rFVIIa infusion was not repeated, given the well-known reported risk of thromboembolism. ROTEM® was measured before the rFVIIa infusion and thereafter, immediately before surgery, as reported in Fig. 2. The Clotting Time(CT) of EXTEM and FIBTEM (extrinsic pathway) decreased from 74 to 45 s and from 76 to 44 s respectively (delta reduction, 39% and 42%). All the ROTEM® values were within the ranges of normality, both before and after the administration of rFVIIa.

## Discussion

ROTEM® did not detect any abnormal profile in our patient at baseline and after administration of rFVIIa, referring to the standard ranges, despite markedly decreased FVII activity [18].

Data from a study on elective neurosurgical patients indicate that the correlation between EXTEM-CT and PT might be weak, at best [19]. However, this study did not consider patients with underlying coagulopathy, who thus remain uncovered from the perspective of evidence. On the other hand, other reports suggested that TEG/ROTEM® could be able to detect the underlying coagulation abnormality, guiding the treatment. In a case series, one out of three FVII deficient patients undergoing surgery showed an increased EXTEM-CT [12], normalized after rFVIIa infusion. Unfortunately, in this report, the effect of rFVIIa infusion on ROTEM parameters was not measured in the two patients who started with a normal baseline. In two reports on FVII deficient parturient women, thromboelastography did reveal an abnormal coagulation pattern, directing the therapy [20, 21]. Bortman et al. reported the safe placement and removal of a cerebrospinal fluid drain placement in a FVII deficient patient using TEG for monitoring the RT [22]. In our report, the parameters related to the extrinsic pathway of coagulation showed a substantial decrease in the Clotting Time, with reductions of 39% and 42% respectively, after the administration of rFVIIa. This suggests a proper haemostasis correction while remaining within the normal range, indicating the effectiveness of rFVIIa in addressing the coagulation issue.

At a first evaluation, compared to the initial hypothesis, it does not appear that the viscoelastic test was superior to conventional tests in defining the risk of perioperative bleeding. However, a significant reduction in CT-EXTEM and FIBTEM probably accounts for the correction achieved with the administration of rFVIIa. The ROTEM® normal ranges are quite broad and do not include specific references for individuals with FVII deficiency. Furthermore, it is possible that using TEG we would have obtained different data. TEG is indeed more sensitive to coagulation abnormalities as it uses different (and generally weaker) activators, resulting in longer reaction/clotting times [23]. It is reasonable to speculate that different threshold values of ROTEM parameters could be identified depending on the type of surgery and the type of coagulation factor deficiencies, but currently, the literature does not provide answers.

Conversely, a negative ROTEM® could also suggest a physiologic coagulation performance in whole blood of this specific patient, despite the severe deficit in a single factor, as underlined by his negative history for bleeding episodes. Based on the largely anecdotal literature on the use of viscoelastic tests [24], the prophylactic administration of RT appeared to be the safest option in this neurosurgical patient.

## Conclusion

Whether this difference between ROTEM® and standard tests is due to the inadequacy of thromboelastographic normal ranges in this setting, or to the absence of clinically significant coagulopathy, has yet to be clarified. ROTEM® is a technique that is increasingly being developed, and practitioners are expanding its indications for use beyond the traditional settings of trauma and consumptive coagulopathy in active bleeding. Its use in perioperative medicine for elective patients currently lacks strong evidence in the literature, particularly for neurosurgical patients [8]. Moreover, in the specific case of rare diseases, the use of this technique is essentially anecdotal. Although we are aware that this work suffers from the intrinsic limitations of all case reports, we believe it provides an opportunity to share interesting data that could be included in a broader context.

## Data Availability

No datasets were generated or analysed during the current study.

## Declarations

### Submission statement

The manuscript has not been previously published or under consideration elsewhere. We confirm that all co-authors have contributed to the clinical management of the case, reviewed the paper and consent to its submission to JCMC.
